# SoftHand at the CYBATHLON: a user’s experience

**DOI:** 10.1186/s12984-017-0334-y

**Published:** 2017-11-29

**Authors:** Sasha Blue Godfrey, Matteo Rossi, Cristina Piazza, Manuel Giuseppe Catalano, Matteo Bianchi, Giorgio Grioli, Kristin D. Zhao, Antonio Bicchi

**Affiliations:** 1 Department of Advanced Robotics, Istituto Italiano di Tecnologia, Genoa, Italy; 2 Research Center “Enrico Piaggio”, University of Pisa, Pisa, Italy; 3 Department of Information Engineering, University of Pisa, Pisa, Italy; 4 Assistive and Restorative Technology Laboratory, Rehabilitation Medicine Research Center, Mayo Clinic, Rochester, USA

**Keywords:** Limb loss, Prostheses, Upper limb, Robotic hand

## Abstract

**Background:**

Roughly one-quarter of upper limb prosthesis users reject their prosthesis. Reasons for rejection range from comfort, to cost, aesthetics, function, and more. This paper follows a single user from training with and testing of a novel upper-limb myoelectric prosthesis (the SoftHand Pro) for participation in the CYBATHLON rehearsal to training for and competing in the CYBATHLON 2016 with a figure-of-nine harness controlled powered prosthesis (SoftHand Pro-H) to explore the feasibility and usability of a flexible anthropomorphic prosthetic hand.

**Methods:**

The CYBATHLON pilot took part in multiple in-lab training sessions with the SoftHand Pro and SoftHand Pro-H; these sessions focused on basic control and use of the prosthetic devices and direct training of the tasks in the CYBATHLON. He used these devices in competition in the Powered Arm Prosthesis Race in the CYBATHLON rehearsal and 2016 events.

**Results:**

In training for the CYBATHLON rehearsal, the subject was able to quickly improve performance with the myoelectric SHP despite typically using a body-powered prosthetic hook. The subject improved further with additional training using the figure-of-nine harness-controlled SHPH in preparation for the CYBATHLON. The Pilot placed 3rd (out of 4) in the rehearsal. In the CYBATHLON, he placed 5th (out of 12) and was one of only two pilots who successfully completed all tasks in the competition, having the second-highest score overall.

**Conclusions:**

Results with the SoftHand Pro and Pro-H suggest it to be a viable alternative to existing anthropomorphic hands and show that the unique flexibility of the hand is easily learned and exploited.

## Background

Benchmarking robotics research can be a challenging task; in some cases, the same lab that developed a novel robotic system has also to create, ex novo, the tasks that are used to evaluate it. In the field of rehabilitation robotics, these difficulties are added to the fact that each device must be tested on individuals, each one with unique characteristics, attitudes, and preferences. A possible solution to this problem can be offered by robotic competitions. Events such as the Robot Cup or the DARPA Robotics Challenge, have proven to be an effective way of benchmarking robotics research and “a driving force of technological development” [1]. In the field of rehabilitation robotics, a unique example is given by the CYBATHLON, which showcases both the abilities of the individual, or “Pilot,” as well as advanced research and commercial technology. It was created “promote the development of useful technologies that facilitate the lives of people with disabilities” in part by “[encouraging] exchange between people with disabilities or physical weaknesses, the research and development world, funding agencies, and the general public” [2].

Limb loss has major effects on various aspects of daily life. A vast number of activities of daily living (ADLs) are dependent on hand function, making upper limb loss particularly devastating for functional independence and ultimately quality of life [3]. Current upper-limb commercial options fall largely into three categories: cosmetic, body-powered, and externally-powered (typically myoelectric) prostheses. Cosmetic prostheses, as the name implies, have a preliminary aesthetic function and only limited functional use, such as a stabilizer or opposition post. This aesthetic function can be an important factor in psychological well-being but may not be sufficient for all users [4]. Research shows these prostheses are primarily used at social events [5], and the main reason cited for rejection is lack of functionality [6]. In contrast, body-powered prostheses (BPPs), operated by means of a cable control system, offer a more functional replacement. These prostheses offer several advantages: a tightly-fitting socket is not as critical as in a myoelectric prosthesis (and typically cushioning material can be used), the device is durable, and the training/learning time is short [7]. Furthermore, a body-powered design allows performance of heavy work in punishing environments that include exposure to dirt or liquids [8]. An important disadvantage of BPPs is that wearers may need to make abnormal movements of the shoulder or wrist in order to operate their prostheses. These movements, called compensatory motion [9, 10], and the discomfort they cause have been cited among the main factors influencing prosthesis abandonment [11]. Furthermore, a person that is unable to generate sufficient force may not be able to operate a body-powered prosthesis. This consideration is particularly true for individuals with limb-loss that prefer to have an anthropomorphic terminal device: body-powered hands require much higher force at the shoulder to activate the prosthesis in comparison to body-powered hooks. Because of the difficulty of use and weak grip, many individuals with amputation reject body-powered hands [5]. Body-powered hooks, however, may be aesthetically objectionable to some users, particularly while adjusting to loss of limb, but are ultimately preferred over body-powered hands because they are lighter and easier to use [12]. In myoelectric prostheses (MPs), the movement is generated by actuators that are powered by a battery and controlled using electromyographic (EMG) signals from the muscles of the residual limb. These prostheses can be operated with minimal effort from the user with respect to BPPs. Though compensatory motion is still seen in users of MPs, it is often less-pronounced because the control is provided by the ipsilateral arm rather than involving the contralateral side. However, MPs are far from being a valid substitute to their older counterparts [13]. MPs are, in fact, less robust than BPPs, and therefore less suited for heavy work or hostile environments. MPs are generally heavier than BPPs, in which the harness also plays a role in partially unloading the socket from the prosthesis weight. Furthermore, fitting, training, and maintenance of MPs results in much higher costs for the user [11]. Finally, EMG control can sometimes be counterintuitive and difficult to master. This final consideration is particularly dependent on the individual: length of the residual limb, time since amputation, and other factors all play important roles in one’s ability to learn and effectively use myoelectric control. As Carey et al. showed in their systematic review of the literature [7], MPs and BPPs have different performance depending on the specific domain, but, overall, each type does not provide a significant general advantage over the other. This phenomenon can also be seen in the similarly high rejection rates for MPs and BPPs (23% and 26%, respectively) [14], showing that more work is needed to provided functional and satisfactory upper limb prosthetic aids.

This work describes the efforts of team SoftHand Pro leading up to and competing in the Powered Arm Prosthesis Race in the CYBATHLON rehearsal and CYBATHLON 2016. Further, we describe how the experience has driven the development of the SoftHand Pro-H, which combines the advantages of BPPs and MPs. In particular, we present our Pilot, the prosthetic devices used in competition, training efforts, and final performance in the two events compared to the rest of the competitive field.

## Methods

A single subject participated in lab testing of novel prosthesis prototypes to prepare for the CYBATHLON rehearsal and 2016 events. Prior to his participation in prototype testing, the training was approved by the regional Ethics Committee and he signed an informed consent. The ethical approval and device risk analysis was reviewed by the organizing committee of the CYBATHLON before being granted permission to participate in competition.

The subject had a unilateral (right), transradial amputation at 14 years of age; he was left-hand dominant prior to amputation. At the CYBATHLON rehearsal, he was 27 years old; at the time of the CYBATHLON 2016, he was 29 years old. The subject used a body-powered hook prosthesis in his daily life.

### Study device

Because this work covers the use of the SoftHand prosthesis in various environments over time, modifications to both hardware and control methods were employed. The SoftHand prosthesis design and control are described in brief below, including these modifications. The SoftHand Pro (SHP, myoelectrically controlled) was used at the CYBATHLON rehearsal, while the SoftHand Pro-H (SHPH, controlled via shoulder harness) was used in the CYBATHLON 2016.

#### SoftHand Pro

The device presented in this paper is the prosthetic version of the Pisa/IIT SoftHand [15], an anthropomorphic hand with 19 degrees of freedom (DOFs) and one degree of actuation. The hand consists of a group of rolling joints connected by elastic ligaments that make the system soft and safe. A single tendon runs through the entire hand, enabling the overall system to adapt during the grasp. The SoftHand is also very robust and can withstand severe joint dislocations and disarticulations. These characteristics, namely design simplicity, adaptability, resilience to high forces, and robustness and reliability make the SoftHand an ideal starting point for a prosthetic device.

**Fig. 1:**
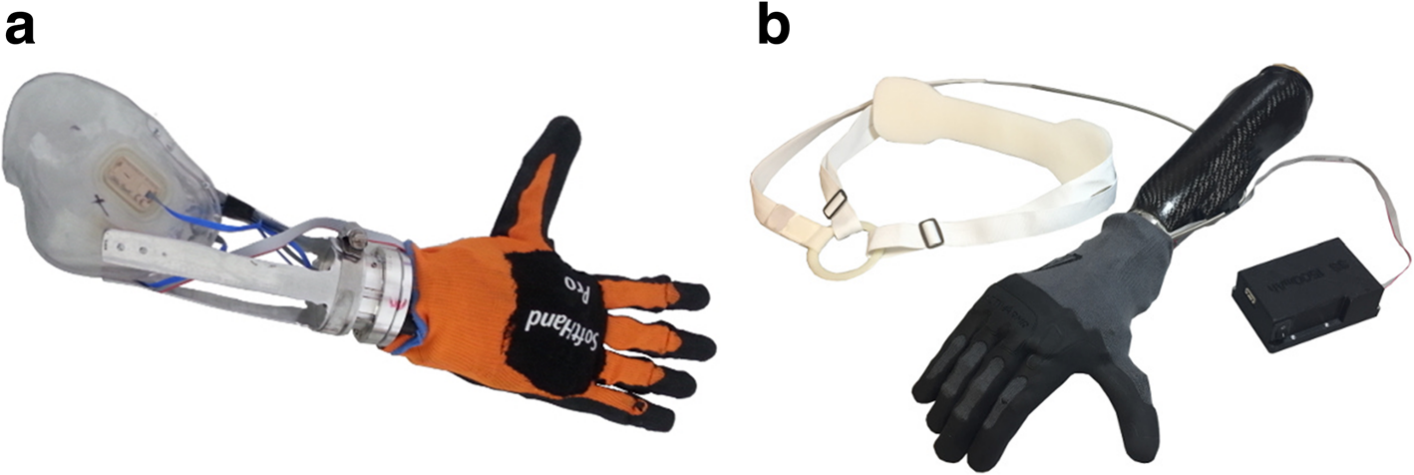
CYBATHLON rehearsal and 2016 Devices. The SoftHand Pro (**a**), used in the rehearsal, was myoelectrically controlled and the SoftHand Pro H (**b**), used in the CYBATHLON 2016, was controlled using a figure-of-nine shoulder harness

The same principles of the SoftHand were translated in a prosthesis prototype called SoftHand Pro, shown in Fig. 1 a. The SoftHand Pro is controlled using commercial surface electromyography (EMG) electrodes (Otto Bock, Germany). These sensors detect the electrical activity from the user’s arm muscles, making it possible to control the hand by applying appropriate muscle contraction. The SoftHand Pro can be easily controlled using two-site myocontrol, as the movement trajectory is flexibly dictated by human synergy patterns, while its adaptivity and flexibility allow it to conform to a wide variety of object shapes and sizes [16].

#### SoftHand Pro-H

The SoftHand Pro-H has the same basis as the SoftHand Pro, and thus also has a single degree of actuation to drive the 19 DOFs and mold the hand around objects it encounters and environmental constraints. The SHPH, however, is controlled using an input lever directly connected with the Bowden Cable of a commercial figure-of-nine harness (Otto Bock, Germany). The SHPH was used in competition as a voluntary-open device, matching the control of the Pilot’s typical body-powered prosthesis; in further development of the SHPH, the ability to easily switch between voluntary-open and voluntary-close modalities was developed and is undergoing testing. The complete setup is shown in Fig. 1 b. This mechanism allows the translation of the Bowden Cable movement into a position-controlled motor command, with a considerable reduction of effort for the user compared to typical BPPs. Through use of the figure-of-nine harness, the Pilot can infer the aperture of the hand via proprioception of the displacement of the shoulder. As mentioned in Table 1, the force required to activate the hand ranges from 3.3 to 6.7 N. The excursion required is adjustable up to a maximum of 18 mm; in the CYBATHLON, the Pilot preferred an excursion of roughly 8 mm. The input mechanism, the motor and the electronic hardware are directly placed on the dorsal part of the hand. Finally, the SHPH also features a wrist interface compatible with existing commercial sockets.

**Table 1:** Specifications for the SoftHand Pro and SoftHand Pro-H. *Activation force applies only to the SoftHand Pro-H

Specification	SoftHand Pro & Pro-H
Weight	520 g
Length	200 mm
Width	90 mm
Maximum Aperture	120 mm
Pinch Grip Force	20 N
Power Grip Force	up to 76 N
Closure Time	1 s
Activation Force*	3.3 - 6.7 N

### Training and testing procedure

To prepare for the CYBATHLON events, the subject trained in the lab for roughly one week on three occasions: once immediately preceding the CYBATHLON rehearsal, once roughly 6 months before the CYBATHLON 2016, and finally immediately preceding the CYBATHLON 2016. The subject had previous limited exposure to the SoftHand Pro approximately six months prior to the CYBATHLON rehearsal. The training utilized common objects as well as simulated versions of tasks from the Powered Arm Prosthesis Race. Each training session began with basic prosthesis control, working from opening and closing the prosthetic hand to completing ADLs. Once the Pilot was comfortable with the device, the training focused on the CYBATHLON tasks. To minimize mental fatigue, the Pilot would attempt a single task two to three times, aiming to familiarize himself with the task, develop strategy, and ultimately improve completion time after which he would focus on a different task. The training rotated through the various tasks in this manner. Intermittently, the Pilot performed the entire simulated course (all six tasks).

### Powered Arm Prosthesis Race

**Fig. 2:**
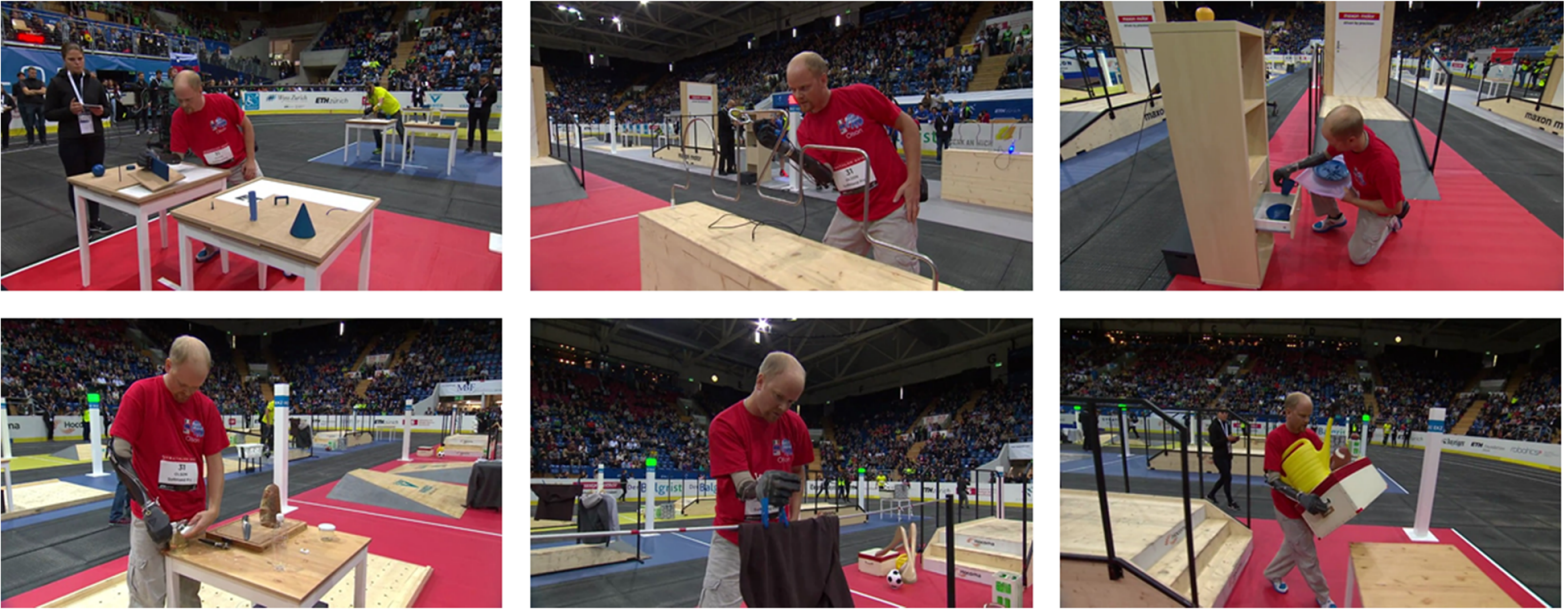
CYBATHLON 2016: Tasks. The figure shows each of the six CYBATHLON tasks as the SHP team Pilot performed them in the finals of the CYBATHLON 2016. The top row, from left to right, shows the Puzzle, Wire Loop, and Shelf and Tray tasks. The bottom row, from left to right, shows the Breakfast Table, Hang-up, and Carry tasks

In the Powered Arm Prosthesis Race, pilots equipped with upper limb prostheses were asked to perform as many tasks as possible in the shortest time possible. The Pilot finished the race as soon as he completed all six tasks or, alternatively, once the time limit was met. The tasks are related to ADLs and were designed to cover the variety and complexity of the challenges that individuals with upper-limb loss face in everyday life. Although the race consisted of the same tasks both at the CYBATHLON 2016 and at the CYBATHLON rehearsal 2015, some rules were changed; therefore it is not possible to make a direct comparison between the two events for most tasks. On a general level, the scoring system, that discouraged pilots to even attempt some of the tasks during the rehearsal, was changed in order to encourage them to complete all of the tasks. On a more specific level, some tasks were changed after the feedback received during the rehearsal. In the CYBATHLON rehearsal, four teams competed in the qualifying and final rounds. In the CYBATHLON 2016, the field of ten competing teams (out of twelve registered teams) was whittled down following a series of qualifiers into an “A” and a “B” final. The teams competing in the A final were ranked between first and fourth place while those in the B final ranked between fifth and eighth. In this section we describe the tasks that compose the most recent Powered Arm Prosthesis Race, highlighting the differences with the tasks that were performed during the rehearsal. A more detailed description of the Powered Arm Prosthesis Race can be found at [17]; a snapshot of each task can be seen in Fig. 2, with the first three tasks in order from left to right in the top row and the last three tasks on the bottom row.

#### Task 1: puzzle

The task was to transfer a 3 x 3 grid of square wooden bases, each with differently shaped “handles” from one puzzle frame to another. The pieces could only be lifted by the handle and the handle could only be manipulated using the prosthetic terminal device. The handles varied in shape, size, texture, and weight.

*Rehearsal:* Instead of being positioned on two different tables with a 0.26m gap in between them, the frames were adjacent one to another. This allowed pilots to drag bases from one frame to the other, without necessarily lifting them completely.

#### Task 2: wire loop

The task was to move a wire loop from one end of a metal wire “course” to another. The wire loop was conductive and any contact with the wire course, with the exception of “safe zones” at the start and finish, resulted in task failure. The course contained 90° turns, diagonal turns, and curves, and the wire loop could be guided only by the prosthetic arm.

*Rehearsal:* The inner diameter of the wire loop was 42mm instead of 75mm, and the base of the wire course was positioned 0.1m higher. This made the task more difficult.

#### Task 3: shelf and tray

At the start of the task, many items used to set a breakfast table were arranged on a set of shelves or in drawers. A tray was also provided. Many of these items could only be handled with the prosthetic device. It was required that all of the items, including the tray, be carried over a ramp, through a closed door, down a ramp and set on a table. The Pilot was allowed as many trips as needed. Finally, one of the items was a lightbulb in a box, which had to be removed from the box and screwed into a table lamp using only the prosthetic device.

*Rehearsal:* More items needed to be carried to the breakfast table; however, all of the items were positioned on shelves and not in drawers, which often allowed the pilots to drag them onto the tray, instead of grasping them. Also, the task of screwing the lightbulb into the table lamp was not present.

#### Task 4: breakfast table

Several elements of meal preparation were set on a table. This task could be completed using either hand/arm for any part of the task. The components of the task were opening a water bottle, opening a jar, unwrapping a sugar cube, cutting a loaf of bread, and using a can-opener to open to a can.

*Rehearsal:* This task remained unchanged from the original rules used in the rehearsal.

#### Task 5: hang-up

A clothesline was set up next to uneven terrain. On the clothesline were two clothes hangers and two clothespins. Nearby, was a hamper with a t-shirt, button-up blazer, and zip-up jacket. The Pilot had to pin the shirt to the line, manipulating the pins with his prosthetic arm only, and close and hang both jackets using the hangers (either or both arms could be used for the jackets).

*Rehearsal:* The task consisted only in pinning six rectangular shaped pieces of thin foam to the clothesline.

#### Task 6: carry

At the start of this task, objects of various sizes and weights were placed near the bottom of a 3-step staircase. The Pilot had to carry the objects up the stairs, over flat ground, down stairs and place them on a table. The Pilot could make as many trips as desired. Objects included soccer and footballs, watering can, water crate, large box, and large bag ranging in weight from roughly 400 grams to nearly 5 kg.

*Rehearsal:* Only two empty boxes, two empty bags, a football and a soccer ball were used, with a maximum weight of roughly 400 grams.

## Results

Overall, the Pilot performed well with both the SHP and SHPH. Due to the Pilot’s familiarity with body-powered prostheses, he chose to use the SHPH in the CYBATHLON 2016 (as opposed to the SHP used in the CYBATHLON rehearsal).

### CYBATHLON training

**Fig. 3:**
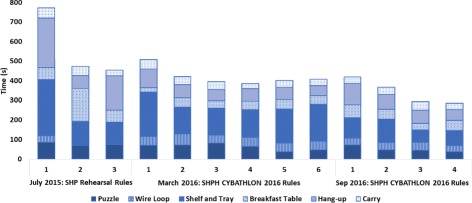
CYBATHLON Training Full Course Results. The figure provides a sample of the results from training for both the CYBATHLON rehearsal and 2016 events, indicating also the device and task rules used. NB: In the first trial, the wire loop was attempted but not completed successfully (time shown)

**Fig. 4:**
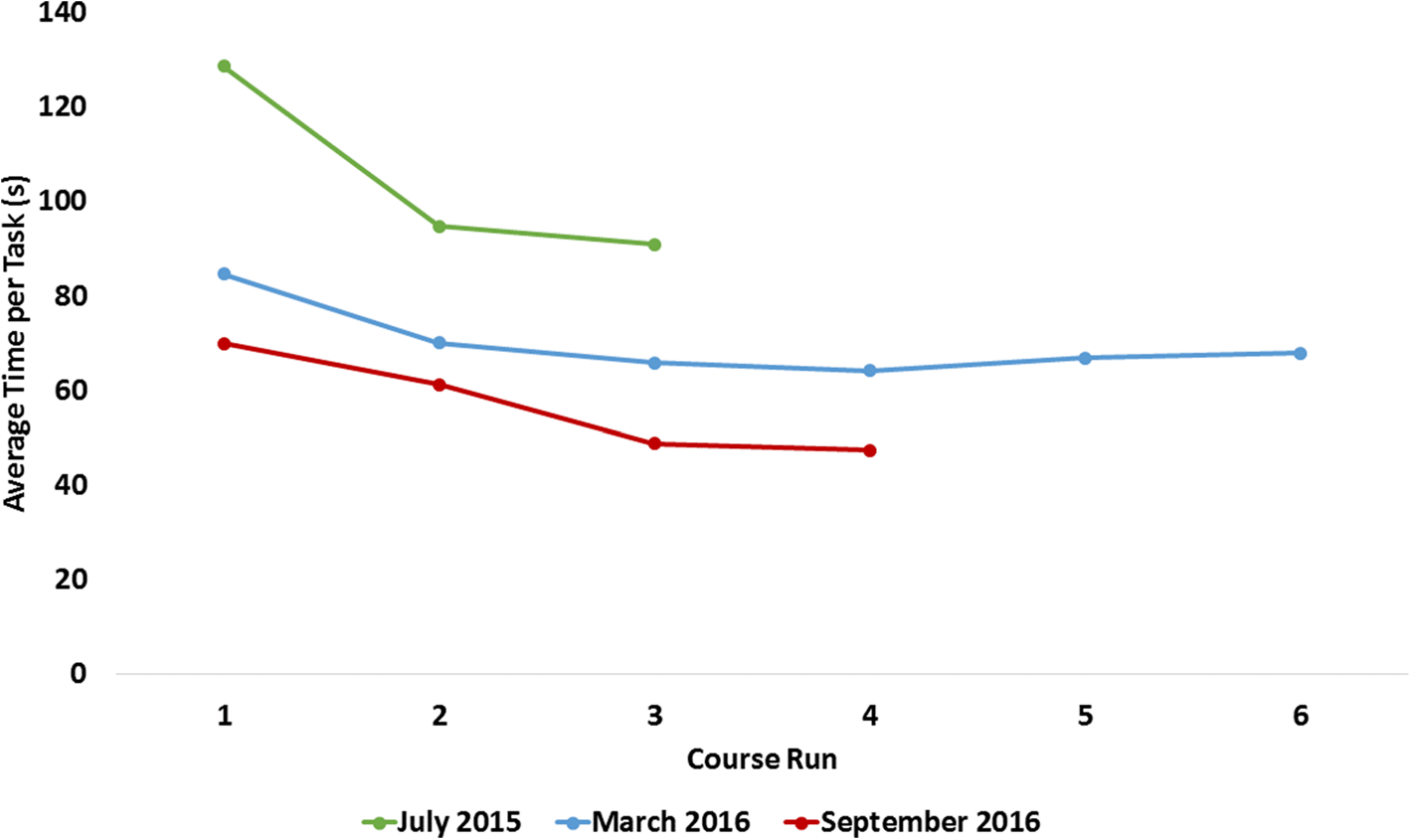
CYBATHLON Training Average Task Results. The figure shows the average time to task completion in the training course runs presented in Fig. 3

As mentioned in Methods, the Pilot had a training session prior to the CYBATHLON rehearsal and two training sessions, roughly six months apart, prior to the CYBATHLON 2016. While the results of each training session cannot be directly compared due to changes in task rules and/or to prosthetic hardware and control methods, overall they show a clear learning phase and plateau. Sample results are presented for each training session in Fig. 3. These results are culled from complete course runs of all six tasks. A “course run” refers to an attempt by the Pilot to complete all tasks in order without pausing. To further understand the effects of training, an average time to task completion was calculated for each complete course run, Fig. 4. The learning curve and plateau are more easily recognized in average time to task completion, in part because the Pilot performed five out of six tasks in the earliest training sessions. Further, the Pilot showed excellent retention of the previous training sessions, as evidenced by the stability in overall time to completion from the end of one training session to the beginning of the next. Rule and/or task design changes produced notable changes in time to completion particularly for three tasks: Wire Loop, Shelf and Tray, and Hang-up. The Pilot had previously chosen to omit the Wire Loop task in competition due to task difficulty (largely due to the Wire Loop’s small diameter in the CYBATHLON rehearsal), whereas following a rule change in which the diameter was increased for CYBATHLON 2016, the Pilot had a high success rate and trained the task to perform in competition. The Shelf and Tray and Hang-up tasks were also changed significantly, as described in Methods. In the former, for the CYBATHLON 2016, the Pilot had to learn to remove cutlery from a drawer organizer and screw in a light-bulb, both of which could only be performed with the prosthetic hand. These task and rule changes can be seen in the large increase in time from the 2015 to 2016 training sessions, and the visible learning curve in the 2016 training sessions. In the latter, the task was changed from using only clothespins to hang up small foam cards to hanging items using clothespins and closing a button-up and zip-up jacket and hanging each with a hanger. Similar to the Shelf and Tray task, the substantial task design change required the Pilot to adopt a new strategy and ultimately resulted in a more stable time-to-completion over the course of training.

**Fig. 5:**
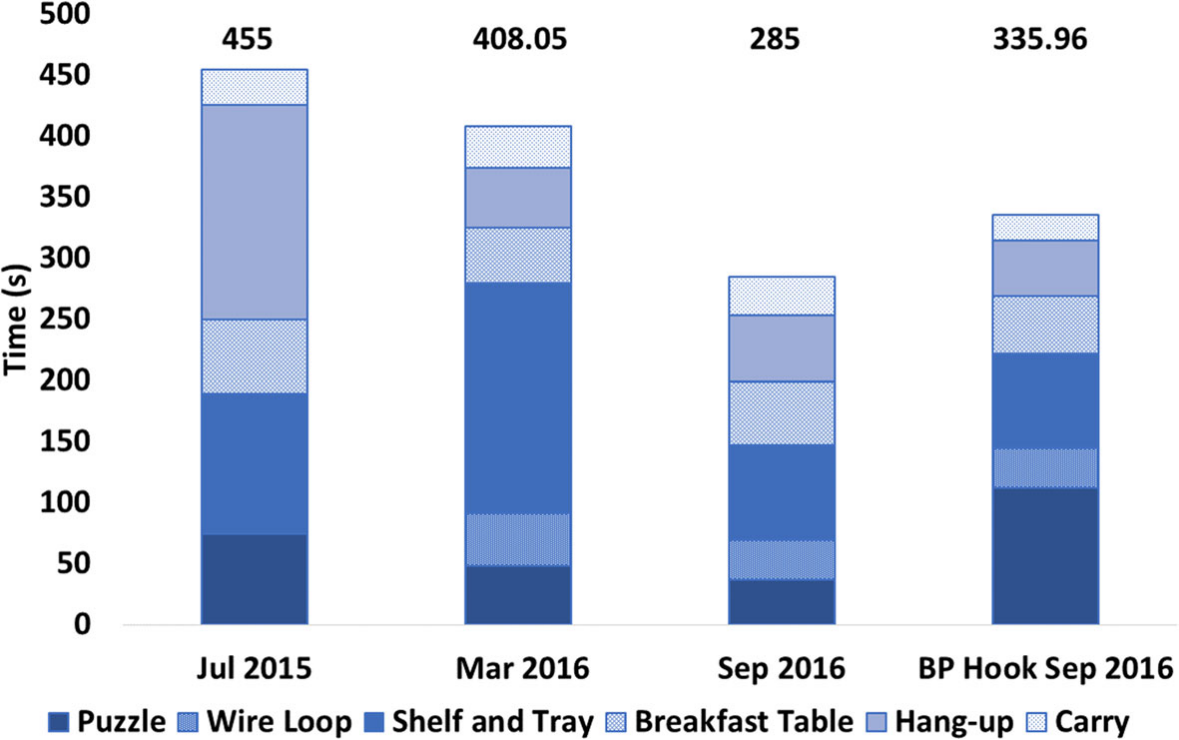
Simulated Course with SHP vs BP. The figure shows the final full course run in each of the three training sessions and a single run completed by the Pilot with his typical prosthesis (a body-powered hook). The total time of each course run (in seconds) is provided above each bar

During the final training session, the Pilot also completed the simulated course once using his typical prosthesis (steel body-powered hook, by Hosmer, USA). A comparison of the final course run in each training session and the attempt with his typical prosthesis is presented in Fig. 5. As the figure illustrates, the Pilot had similar performance in his final training with the SHPH as with his bp hook. Noticeably different, however, was his performance on the Puzzle task: many of the shapes included in the Puzzle task were difficult for him to grasp with the hook, necessitating multiple grasp attempts, often involving compensatory movements.

### CYBATHLON rehearsal and 2016 results

**Fig. 6:**
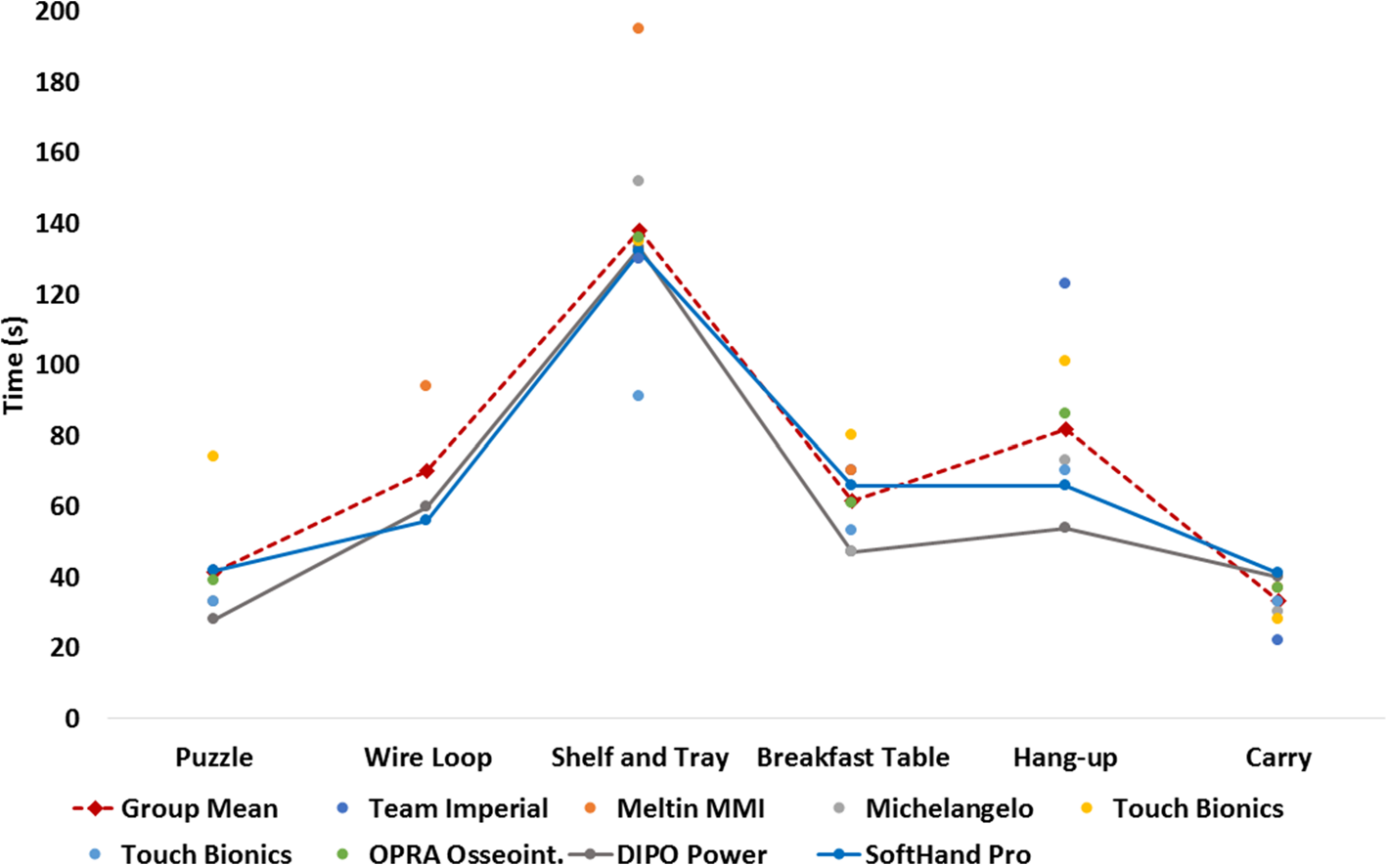
CYBATHLON 2016 Results. The figure shows the results of Team SoftHand Pro and the CYBATHLON winner (Team DIPO Power) alongside the mean results all participants in the final and their individual results. NB: not all competing teams completed all tasks

As previously described, the CYBATHLON event was divided in two phases: qualifications and finals. The tasks were identical in both phases; the first phase served to narrow the field to eight teams. Note: twelve teams were registered in the CYBATHLON 2016; however only ten participated in the competition. In the qualifying round, team SHP placed sixth and thus entered the B final (data not shown). Results from the finals follow: Fig. 6 presents the results of the SoftHand Pro team Pilot as well as the other seven teams competing in the final. Each team’s result in each task completed (not all teams completed all tasks) is indicated by a dot. Further, the group mean, winning team (DIPO Power), and Team SoftHand Pro’s performance are each indicated by a line. While a full, statistical analysis is not appropriate for this type of data, the results indicate that the SHP Pilot’s performance was competitive and in some tasks superior to the group. This performance can be evaluated both in terms of time to task completion as well as in total number of tasks completed within the 8-minute time-limit. Only two teams (including SHP) completed all six tasks. Looking at the tasks individually, all eight teams completed the Shelf and Tray, Breakfast Table, and Carry tasks. Seven teams completed the Hang-up Task and six the Puzzle task. Most challenging was the Wire Loop task, completed by only three teams. Overall, team SHP finished first in the B final, thus 5th out of 12 registered teams, and was the second team (and only robotic device) to complete the full course. Team DIPO Power was the only other team to complete all six tasks, scoring the same number of points as the SHP Pilot and having a faster overall time (completing the course in 362 s compared to SHP’s 403 s). DIPO Power was also the only team to use a body-powered prosthesis (using the TRS Grip 5 prehensor, TRS, USA). Comparing the performance of the two teams, as can be seen in 6, the two teams had comparable completion times for 3 tasks (± 4 seconds; the Wire Loop, the Shelf and Tray, and the Carry task), while team DIPO Power performed an average of 15 seconds better than team SoftHand Pro on the remaining three tasks.

**Fig. 7:**
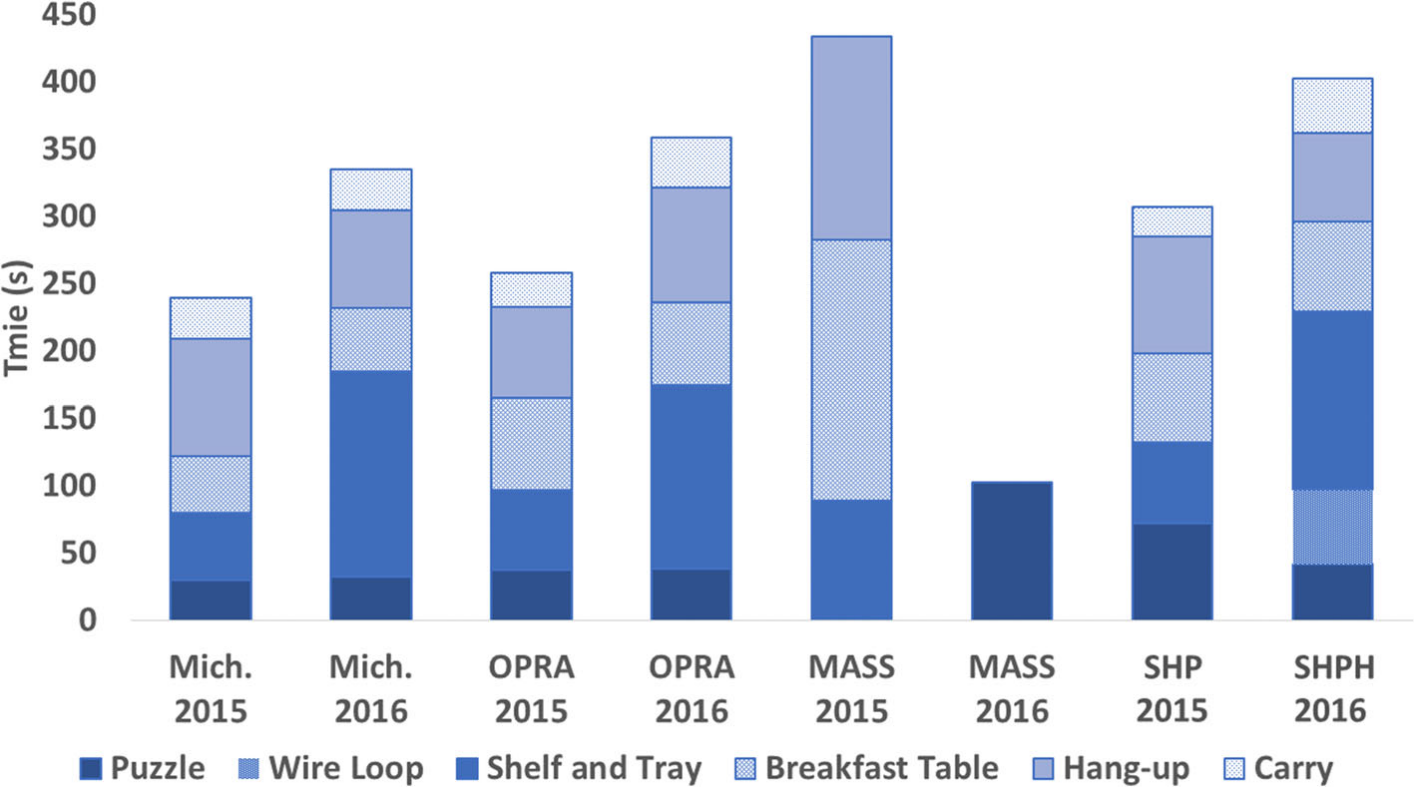
CYBATHLON rehearsal and 2016 Comparison. The figure shows the results of the four teams that participated in both the CYBATHLON rehearsal and 2016 events. The team names have been abbreviated as follows: Mich. is Team Michelangelo; OPRA is Team OPRA Osseointegration; MASS is Team M.A.S.S. Impact; and finally SHP is Team SoftHand Pro. NB: Absence of a particular task indicates the task was either not attempted or failed by the Pilot

As this work presents both the CYBATHLON rehearsal and CYBATHLON 2016 events, Fig. 7 shows the results of the four teams that competed in both events. (NB: The CYBATHLON was open to both research and commercial teams, and teams were allowed to use a combination of research and commercial methods or devices, thus the specifications of the devices and control methods were not apparent or known for all teams. The information on individual teams listed below is culled from the CYBATHLON Team information page and Team webpages or interviews, where indicated, and is accurate to the best of the authors’ knowledge [18]). Team Michelangelo used a Michelangelo hand (Otto Bock, Germany; a commercially available microprocessor hand) with the standard control the Pilot uses in everyday life. Team OPRA Osseointegration used a commercially available tridigit hand (exact model unknown); the prosthesis does not use a traditional socket but rather osseointegration to interface with the user’s residual limb and is controlled via implanted electrodes [19]. Finally, Team M.A.S.S. Impact used a Bebionic3 hand (RSL Steeper, UK; a commercially available microprocessor hand) with a research control method using force myography and pattern recognition [20]. Because, as described in the Methods section, tasks were modified between the CYBATHLON rehearsal and 2016 events, a direct comparison of any team’s performance between the two events is not possible. However, comparing multiple teams’ performances over the two years suggests which changes in performance are due to task changes versus training, hardware updates, or other aspects that directly affect the time of the individual pilot. For example, teams Michelangelo and OPRA Osseointegration showed consistent performance on the Puzzle task whereas team M.A.S.S. Impact and team SHP showed improved performance, likely indicating these changes were due to team-specific changes in training or device. Conversely, the Shelf and Tray task consistently shows a decrease in performance across all teams (longer time to completion or failure to complete), likely due to differences in task complexity. Others were not significantly changed and showed little change in individual performance (namely the Breakfast Table, Hang-up, and Carry tasks), possibly suggesting a floor effect associated with these tasks. Notably, the Wire Loop task was not attempted by any of the four pilots in the rehearsal event because of task difficulty and strict task rules (a single contact between the loop and the wire course results in immediate failure). Among these four teams, this task was only successfully completed by team SHP in the 2016 event.

### User observations

We also conducted an informal interview with the team SHP Pilot to get his feedback on the SHP used in the CYBATHLON rehearsal and the SHPH used in the CYBATHLON 2016. Most of the less-desirable aspects of the system refer to both the SHP and SHPH, which approximate a large male hand (roughly 95th percentile). The Pilot found that the prosthesis weight leads to fatigue and limits intense use; the Pilot also noted that with his existing BP hook, he prefers to use his other hand 65-75% of the time). Similarly, he finds the prosthesis to still be a bit oversized in terms of palm width and possibly hand length. He also mentioned that, because he still needs to integrate both the mass and size into his body schema, using the SHP or SHPH requires more focus than his hook. The Pilot was pleasantly surprised by both the low force and small excursion required to activate the SHPH, remarking that it rendered the harness much more comfortable. Further, he found the grasp pattern on the SHPH very helpful, noting it provided him with more confidence, and he liked the flexibility of the fingers, which allowed him to be more free with his movements. Similarly, he appreciated the rubber grip of the glove and the addition of the fingernails, which “increase the versatility and function of the hand.” Finally, he greatly enjoys being able to maintain and modify his existing prosthesis (eg: improving the grip with athletic tape, changing the elastic bands, and making attachments such as for a rifle light) and saw learning to reposition dislocated fingers on the SHP or SHPH in a similar light.

## Discussion

The CYBATHLON provided a unique experience to test the novel SHP and SHPH prostheses in a rigorous and competitive environment alongside both research and commercial prosthetic systems. The Pilot performed well with both devices, and the experience helped illustrate the advantages and disadvantages of the terminal device and different control modes used. In the CYBATHLON rehearsal, the Pilot placed third out of four competing teams; the three other teams all competed using commercial prosthetic hands, although two of these used a research control method. During the training session, the Pilot became proficient in myoelectric control. However, fatigue was a real challenge that over time lead to increased co-contraction of the muscles controlling the prosthesis and thus reduced performance. This was likely due to a combination of factors: First, the Pilot had relatively little overall exposure to myoelectric control, and muscle fatigue is to be expected in new users of myoelectric devices; it is conceivable that with daily use, the Pilot would have been able to use the device longer without suffering fatigue. Second, the myoelectric socket and SHP are heavier than the Pilot’s typical prosthesis and do not have the potential off-loading provided by the shoulder harness. Further, the myoelectric socket requires a more snug fit and, in order to ensure good contact with the electrodes, does not allow the possibility of using a prosthetic sock. The myoelectric socket for the Pilot, who has a relatively short residual limb, also limited elbow range of motion and created some discomfort over time due to the increased pressure.

Despite performing well in the CYBATHLON rehearsal, the Pilot’s familiarity with body-powered prostheses and above-mentioned challenges with myoelectric control served as an inspiration to adapt the SHP to alternative control methods. With the SHPH, the Pilot was able to use a control method he was familiar with (figure-of-nine shoulder harness), while benefiting from certain advantages of a powered prosthesis. Namely, the powered prosthesis reduced the load on the shoulder, allowing him to use minimal force to activate the prosthesis. Further, the excursion distance required of the shoulder could be adjusted to his comfort level, balancing movement and control sensitivity to the Pilot’s preference. Note: the SHPH has a maximum excursion of 18 mm (for comparison, an Ottobock hook measured in the same way (linear distance of the activation lever) has an excursion of 44 mm). At the start of training, the Pilot used an excursion of roughly 15 mm, but throughout the training experimented with smaller excursions, using approximately 8 mm for the competition. This distance provided sufficient and satisfactory resolution for the Pilot and can be customized to the preferences of the user to ensure maximum comfort and usability. Finally, the SHPH provided an anthropomorphic terminal device without the mechanical load incurred when using a body-powered hand, thus minimizing discomfort associated with the harness. The Pilot used the SHPH as a voluntary-open terminal device in competition; however, the device can also be easily switched to voluntary-closed to allow greater versatility and comfort.

At the start of training for the CYBATHLON 2016, the Pilot tested both the SHP and SHPH. He was given time to refresh his memory and practice with the SHP as well as time to familiarize himself with the SHPH. The Pilot chose to use the SHPH in competition because he was more comfortable and experienced with the control method and thus thought it would lead to more reliable and consistent performance. This comfort with the shoulder harness control may be an important contributor to the apparent retention/carry-over from the first training session with the SHP to the first training session with the SHPH. Although it is difficult to estimate the exact impact, it is also important to note that there were task changes as well as hardware changes between sessions. Within each session, though, the improvement in performance over different trials suggests using that the SoftHand terminal device is intuitive and easy to learn.

In the qualifying round of the CYBATHLON 2016, the Pilot did not complete the Wire Loop task successfully and then struggled in the Breakfast Table task. Though he completed the Breakfast Table task, he did not have enough time to complete the final two tasks, resulting in him entering in the B final. It is important to remember that CYBATHLON Pilots were not necessarily trained competitors accustomed to the stress of competing in a large arena and that the simulated tasks in the lab could not replicate exactly the race course. Because of the scoring method employed, typical also of other sporting events, the Pilot could not rank above fifth place by competing in the B final. Team SHP was one of two teams, and the only team with a robotic hand, to complete all six tasks in the final and had the second highest score (a result of both number and type of tasks completed as well as overall time to completion). As mentioned above, the winning team performed an average of 15 seconds faster on three tasks (the Puzzle, Breakfast Table, and Hang-up tasks). While it is difficult to parse out precisely what contributed to these differences, one likely aspect is that these tasks benefited from the precise and reproduceable grasp of the TRS Grip 5. The SHP’s and SHPH’s flexible, adaptable design may require more training in order to be efficient in certain tasks, as expanded on in the discussion below. The robustness and functionality of the design, however, is demonstrated by the fact that the SHP Pilot was the only other Pilot able to complete all 6 tasks, 3 of which were completed only a few seconds faster or slower than Team DIPO Power.

As mentioned above, the CYBATHLON rehearsal and 2016 events furnished an opportunity to strenuously test the prosthetic system in competition. The experience served to gain insight on strengths and weaknesses in the SHP/SHPH design that can be improved upon in the future. To the authors’ knowledge, no commercial prosthetic hands are flexible. The fingers of the SoftHand Pro and Pro-H can bend out of the way in the event of a collision or simply in response to environmental constraints. Through the CYBATHLON events and trainings, we saw evidence of how this feature can be an advantage but, likely because it is a departure from typical prosthetic design, must be tempered by functional training. For example, the flexibility of the fingers, allowed the Pilot to grasp the handle between his index and middle or middle and ring fingers in the Wire Loop task. Positioning the handle in this way minimized the shoulder compensation necessary to complete the task, even without having an active prosthetic wrist, thus facilitating successful task completion. In contrast, a typical rigid prosthetic grasp could have made the initial approach to picking up the cutlery in the Shelf and Tray task more straightforward. In the training sessions, the Pilot initially struggled to pick up the flat cutlery, in particular the knife, from inside the drawer organizer. However, with training, he learned to use the SHPH’s flexible fingers to his advantage. Further, this experience inspired us to add nails to the design, helping pry flat objects from a flat surface.

### Lessons learned

The experience of preparing for and competing in the CYBATHLON rehearsal and CYBATHLON 2016 illuminated several benefits as well as potential pitfalls of this kind of competition. The involvement of all members of the team in training and development enabled a recursive design process with the user in the loop that lead to many improvements in the SHP/SHPH and their control. One example of this is the introduction of the fingernails, which greatly improved grasping capacity in specific tasks, and were borne out of repeated observation of the Pilot during the training sessions, attempts at different strategies to accomplish the tasks, and brainstorming among the team. This iterative, user-centric design process is useful both for research and commercial development as well as in clinical practice. Much in the way that prosthetists tailor prosthetic solutions for the individual, the design and development process must always strive to keep the user’s needs in mind and test these solutions throughout the design process with end-users. One aspect of the CYBATHLON that can be both a potential boon to technology development as well as a potential pitfall is the restriction against using the contralateral hand in many tasks or task aspects. Many individuals with unilateral amputations use their sound hand for more delicate or dextrous tasks. This restriction in the CYBATHLON can limit the naturalness of the Pilot’s actions. Similarly, it can encourage the development of technology specifically to beat task challenges rather than to tackle everyday, real-world problems. However, this approach may also push the boundaries of available technology and inspire the development of new prosthetic solutions that could indeed be relied on for a wider variety of tasks. A parallel to this situation can be seen in the results of the training. In training, the Pilot not only needed to learn to use the SHP and SHPH but also to complete the tasks in the most strategic way for the competition. Because the CYBATHLON is a competition and each task has specific rules, finding the best strategy did not always consist of the most logical or aesthetically pleasing set of movements but rather favored speed. As mentioned for technological development above, this constraint forced the Pilot to find new ways of accomplishing tasks with the prosthetic hand for which he would have typically relied on his sound hand. While not all of these will likely carry over to his daily life, some of them likely will, and the training session may encourage him to continue experimenting and exploring new approaches to incorporating the use of his prosthesis in his everyday life.

### Current and future work

The SHP Pilot’s comments related to size and weight of the SHP and SHPH systems fall in line with current work being performed to improve the SoftHand Prosthesis line. In particular, reducing both hand and battery size and weight by reducing motor power as well as designing a smaller hand size approximating that of an average female is a current research aim. Further, the SHP and SHPH systems will allow future research to isolate the effects of the control mode in comparison testing. Similarly, and as mentioned above, the excursion required to actuate the SHPH can be customized to the user, thus further tests are required to establish the effects of this excursion on shoulder compensatory motion and control capacity.

## Conclusion

Overall, the SHP and SHPH prosthetic systems proved strong competitors in an international competition pitting research and commercial upper-limb prosthetic systems against each other to complete both abstract tasks and those based on activities of daily living. At the CYBATHLON, pilots found themselves in a competitive context that pushed them to refine movements with their prosthetic hands, while in most cases restricting or eliminating use of the contralateral hand. This context was the fertile soil in which the SoftHand Pro team was able to crack the glass wall between two pre-existing MP and BP paradigms and build and test a new prosthesis that took advantage of the strengths of both. The experience of the CYBATHLON has shown the feasibility of a flexible, synergy-based, and anthropomorphic prosthetic hand.

## Abbreviations

ADL: Activities of daily living
BPP: Body-powered prosthesis
DOF: Degrees of freedom
EMG: Electromyography
MP: Myoelectric prosthesis
SHP: SoftHand Pro
SHPH: SoftHand Pro-H
